# Sexual function in women with endometriosis and pelvic floor myofascial pain syndrome

**DOI:** 10.61622/rbgo/2024rbgo40

**Published:** 2024-05-27

**Authors:** Rayanne Moreira da Cunha, Mariana Oliveira Veloso, Samuel Soares Coutinho, Luana Darc de Menezes Braga, Adriana Silva de Barros, Germana Mesquita Magalhães, Pedro Olavo de Paula Lima, Simony Lira do Nascimento, Leonardo Robson Pinheiro Sobreira Bezerra

**Affiliations:** 1 Universidade Federal do Ceará Fortaleza CE Brazil Universidade Federal do Ceará, Fortaleza, CE, Brazil.

**Keywords:** Endometriosis, Pelvic pain, Pelvic floor, Muscle tonus, Trigger points, Myofascial pain syndromes, Sexual satisfaction

## Abstract

**Objective:**

To evaluate and compare the sexual function and pelvic floor muscles (PFM) function of women with endometriosis and chronic pelvic pain (CPP) with and without Myofascial Pelvic Pain Syndrome (MPPS).

**Methods:**

Cross-sectional study conducted between January 2018 and December 2020. Women with deep endometriosis underwent assessments for trigger points (TP) and PFM function using the PERFECT scale. Electromyographic activity (EMG) and sexual function through Female Sexual Function Index (FSFI) were assessed. Statistical analyses included chi-square and Mann-Whitney tests.

**Results:**

There were 46 women. 47% had increased muscle tone and 67% related TP in levator ani muscle (LAM). Weakness in PFM, with P≤2 was noted in 82% and P≥3 in only 17%. Incomplete relaxation of PFM presented in 30%. EMG results were resting 6.0, maximal voluntary isometric contraction (MVIC) 61.9 and Endurance 14.2; FSFI mean total score 24.7. We observed an association between increased muscle tone (*P*<.001), difficulty in relaxation (*P*=.019), and lower Endurance on EMG (*P*=.04) in women with TP in LAM. Participants with TP presented lower total FSFI score (*P*=.02). TP in the right OIM presented increased muscle tone (*P*=.01). TP in the left OIM presented lower values to function of PFM by PERFECT (P=.005), and in MVIC (P=.03) on EMG.

**Conclusion:**

Trigger points (TP) in pelvic floor muscles (PFM) and obturator internus muscle (OIM) correlates with poorer PFM and sexual function, particularly in left OIM TP cases. Endometriosis and chronic pelvic pain raise muscle tone, weaken muscles, hinder relaxation, elevate resting electrical activity, lower maximum voluntary isometric contraction, and reduce PFM endurance.

## Introduction

Endometriosis is a chronic condition where endometrial tissue grows outside the uterus. It is associated with pelvic pain and infertility.^(1,2)^ Clinical presentations include infertility, painful periods, pain during intercourse, changes in bowel and urinary habits. Additionally, chronic pelvic pain (CPP) is a related condition defined as persistent pain in the pelvic region lasting longer than 6 months, significantly impacts physical, mental, and social well-being.^(3)^ The etiology of CPP involves multiple systems and can be influenced by psychological and sociocultural factors.^(4)^ Musculoskeletal dysfunctions, resulting from trauma or postural changes, contribute to pelvic pain by decreasing range of motion and causing muscle tension to the formation of trigger points (TP).^(3)^ Women with CPP adopt pain-relieving postures that strain the pelvic area, affecting the pelvic floor muscles (PFM).^(5)^ PFM sensitivity changes are prevalent in women with CPP and muscle strains, intensifying symptoms like dyspareunia and constipation.^(4)^

The main alterations in PFM resulting from pelvic pain are muscle spasms and TP, which can contribute to muscle weakness. TP are hypersensitive areas that compromise the ability of the muscle to contract and relax, leading to pain that may radiate.^(6,7)^ Muscle spasms, on the other hand, are involuntary motor responses that can repeatedly stimulate pain receptors and cause local ischemia.^(8)^ Myofascial Pelvic Pain Syndrome (MPPS), characterized by tense and sensitive PFM, is common in conditions like endometriosis and can impact sexual function.^(9)^

It's crucial to recognize that patients with endometriosis may encounter pelvic floor dysfunction, adding to the intricate web of pelvic pain disorders. In these instances, the pelvic floor dysfunction linked with MPPS can present as symptoms such as dyspareunia, constipation, bladder pain, and vulvar pain, which are common issues reported by individuals dealing with endometriosis. In essence, endometriosis can disrupt sexual function by inducing pelvic pain, particularly dyspareunia. The complex connection between endometriosis, pelvic pain disorders, and sexual function underscores the significance of adopting a comprehensive, multidisciplinary approach to both diagnosis and management.^(9,10)^

Given the complex interaction between pelvic pain, dyspareunia, and involvement of musculoskeletal and central nervous systems in women with endometriosis and CPP, medication and surgical approaches may not be sufficient to improve symptoms and improve the quality of life for these women.^(11)^ Therefore, the purpose of the current study is to evaluate and compare the sexual function and pelvic floor muscles (PFM) function of women with endometriosis and chronic pelvic pain (CPP) with and without Myofascial Pelvic Pain Syndrome (MPPS).

## Methods

This was a cross-sectional observational study with prospective data collection, conducted at the Clinical Research Unit of the Federal University of Ceara' Hospital complex and at Maternidade Escola Assis-Chateaubriand (MEAC), from January 2018 to December 2020.

The sample was of the non-probabilistic type by convenience, composed of women diagnosed with deep endometriosis and CPP seen at the Endometriosis and Pelvic Pain Outpatient Clinics. Sample size calculations were not performed in any analyses within the study. Patients were recruited from the Gynecology outpatient clinic of the research institution and invited to participate in the study by members of the multidisciplinary team, including medical professionals and physiotherapists. Initially, we enrolled 50 women, but four were excluded due to missed the assessment, totaling 46 participants. All participants provided their signature on the informed consent form. Inclusion criteria were women between 18 and 45 years of age with a standard clinical and imaging diagnosis by ultrasound or magnetic resonance of deep endometriosis who had CPP. Exclusion criteria included cognitive dysfunction, presence of vulvodynia or vaginismus diagnosis, postmenopausal status, spinal surgery, pelvic surgery, history of urinary tract infections, neurological disease, herniated spinal disc and symptoms suggestive of overactive bladder or interstitial cystitis/painful bladder syndrome.

Data collection involved screening the patients using a checklist, obtaining sociodemographic and clinical information, evaluating the pelvic floor muscles (PFM), and administering the Female Sexual Function Index (FSFI) questionnaire. PFM assessment included inspection, vaginal palpation, and electromyography.

The PFM evaluation was performed by one experienced physiotherapist in diagnosing pelvic floor dysfunctions. The participant was positioned in modified lithotomy, and the examination was performed through vaginal inspection and palpation. Initially, inspection of vulvar trophism, perineal awareness, use of accessory muscles during PFM contraction, clitoral and cutaneous-anal reflexes, provocative tests for urinary loss and prolapses were performed. Subsequently, unidigital palpation was performed up to approximately the middle third of the vagina to assess PFM tone at rest, palpation of the levator ani muscle (on the right, left, and center through the posterior wall of the vagina) and the right obturator internus (ROIM) and left obturator internus (LOIM) muscles to assess the presence and location of TP.^(12)^ Assessment of PFM function was performed using the PERFECT scale, with P (Power) defined as muscle contraction or pressure capacity during maximal voluntary contraction (MVC) graded from 0 to 5 (modified Oxford Scale), E (Endurance) defined as muscle endurance or time to maintain the MVC in seconds (0 to 10 seconds), with R (Repetition) defined as the number of times the MVC was repeated with the same strength and endurance (0 to 10), F defined as fast contractions (0 to 10), and ECT consisting on timing all steps of the assessment.^(13)^ Muscle function was also classified by the International Continence Society (ICS) scale into strong, normal, weak, or absent. Muscle relaxation of PFM after maximal contraction was classified into complete, partial, incomplete, and absent.^(14)^

Electromyographic activity of the PFM was recorded using an EMG equipment (Miotec® Miotool 400) with the participant in a modified lithotomy position. An endovaginal sensor was manually introduced and positioned on the lateral wall of the vagina, while a reference electrode was placed on the right wrist.^(15)^ All data were transmitted in microvolts (μV) to the equipment software connected to a notebook, making sure that all electrical equipment was disconnected from the power grid during data collection and that the room was adequate to minimize interference. The contraction of the PFM was previously taught in the previous step of the exam (palpation), as well as the orientation to avoid contraction of the accessory muscles during collection. Resting PFM activity was recorded for 60 seconds, followed by three 5-second maximal voluntary isometric contractions (MVIC) with 5-second intervals. The highest value reached during the MVICs was recorded. Additionally, the participant was instructed to sustain the contraction for as long as possible, and the mean values obtained within 60 seconds were recorded.^(15)^

The self-administered Female Sexual Function Index (FSFI) questionnaire evaluated female sexual function across six domains: sexual desire, arousal, lubrication, orgasm, satisfaction, and pain. Composed by 19 questions, scores ranging from 0 to 5 were assigned to each question, and the total score^(2–28)^ was calculated by summing the domain scores multiplied by a factor. Higher scores indicated better sexual function. This approach is also substantiated for women with endometriosis in Brazilian population, identifying the score of 26.5 as an optimized cut-off point to distinguish between normal sexual function and sexual dysfunction.^(28)^ How higher the score, better the sexual function. The FSFI questionnaire was completed in a private room with the assistance of a researcher if needed.

The collected data were stored in the Research Electronic Data Capture (REDCAP) system. Statistical analyses were conducted using the Statistical Package for Social Sciences, version 22.0 (SPSS) and software version R3.3.1. Prior to analysis, the Shapiro-Wilk test was employed to assess data normality, and a significance level of 5% was applied for all tests. Descriptive analysis was performed, utilizing means and standard deviations for continuous variables, and absolute and relative frequencies for categorical variables. The participants were categorized based on the presence or absence of trigger points (TP) in the levator ani muscle (LAM), right obturator internus (ROIM), and left obturator internus muscle (LOIM). Bivariate analysis was conducted to examine the relationships between the dependent variables (PFM functions, electromyographic data, and sexual function) and the independent variable (presence of TP in LAM and OIM). Categorical variables were assessed using the chi-square test, while continuous variables were analyzed using the Mann-Whitney test. It is important to note that there was a loss of 1 participant in the analysis of sociodemographic characteristics and 4 participants in the analysis of the Female Sexual Function Index (FSFI) questionnaire, as a result of inadequate completion of the questionnaires.

The study received approval from the Ethics Committee of the Maternidade Escola Assis-Chateaubriand (registration number 2.189.201 – CAAE: 70799417.2.0000.5050).

## Results

The total 46 women participated in this study and were submitted to PFM evaluation. There was a loss of 1 participant's demographic data. The sociodemographic data are represented in table 1.

**Table 1:** Sociodemographic and clinical characteristics of women with endometriosis and CPP included in the study

Variables		Mean (± SD) n(%)
Age (years)		33.4±6.1
Color		
	White	19(42.2)
	Brown	22(48.8)
	Black	4(9)
BMI Classification		
	Low weight	1(2.2)
	Normal	18(40.0)
	Overwieght	14(31.1)
	Obesity I	12(26.7)
Education		
	Up to elementar school	8(17.8)
	Up to high school	26(57.8)
	Up to higher education	11(24.4)
Marital status		
	Single	16(35.5)
	Married or in a stable union	29(64.5)
	Pregnancy	8(17.8)
	Hormonal contraceptive methods	31(68.9)

BMI - Body mass index

Table 2 presents the findings of the PFM evaluation. The participants did not use accessory muscles (39/46, 84.8%) and had voluntary perineal awareness (25/46, 54.3%), pain on superficial palpation (11/46, 23.9%), bulbocavernosus (39/46, 84.8%) and cutaneous-anal reflexes (37/46, 80.4%), increased muscle tone (22/46, 47.8%) and high frequencies of TP in the LAM and OIMs - being larger on the left in both. Most women had strength deficits and lower PFMs relaxation capacity (partial, incomplete or absent), as well as higher electrical activity at rest and lower MVIC and endurance assessed by electromyography.

**Table 2:** Descriptive analysis of the PFM assessment of women with endometriosis and CPP

Variables		Mean(± SD) n(%)
Perineal awareness		25(54.3)
Use of acessory musculature		7(15.2)
Bulbocavernosus reflex		39(84.8)
Cutaneous-anal reflex		37(80.4)
Pain on superficial palpation		11(23.9)
Vaginal tonus		
	Normal	23(50.0)
	Hypotonic	1(2.2)
	Hypertonic	22(47.8)
	TP in LAM	31(67.40)
	TP in LAM on the right	24(52.2)
	TP in LAM at center	25(54.3)
	TP in LAM on the left	30(65.2)
	TP in ROIM	17(37.0)
	TP in LOID	25 (54.3%)
	P	1.76±0.87
	P ≤2	38(82.6)
	P ≥3	8(17.4)
	E	3.0±3.2
	R	2.4±2.3
	F	3.8±3.4
ICS classification		
	Strong	1(2.2)
	Normal	7(15.2)
	Weak	34(73.9)
	Absent	4(8.7)
Relaxation after maximum contraction		
	Complete	14(30.4)
	Partial	7(15.2)
	Incomplete	14(30.4)
	Absent	11(24)
Relaxation after maximum contraction (category)		
	Complete	14(30.4)
	Incomplete	32(69.6)
Electromyography (μv)		
	Rest	6.0±2.8
	MVIC	61.9±38.7
	Endurance	14.2±10.4

TP - Trigger point; LAM - Levator ani muscle; ROIM - Right obturator internus muscle; LOIM - Left obturator internus muscle; ICS - International Continence Society; MVIC - Maximum voluntary isometric contraction

No participant complained of urinary incontinence and pelvic organ prolapses. When analyzing the variables with the presence or absence of TPs in the LAM, we observed an association between increased muscle tone and the presence of Trigger Points in the LAM (*P*<.001). When analyzing the relationship between the function of the PFMs and the TPs in the LAM, there was no significant difference in the functioning of the PFMs between the women with the presence or absence of the TPs, although a high frequency of muscle weakness was observed in both groups (*P*=.74) (Table 3). In the relationship between relaxation after maximum contraction of PFMs and the presence of TPs in the LAM, a statistically significant difference was observed, in which women with TP represented the highest rate of participants with difficulty relaxing (*P*=.019).

**Table 3:** Comparison of pelvic floor muscles function according to the presence of trigger points in the levator ani muscle in women with endometriosis and CPP.

Variables		TP in LAM present (n=31) n(%)	TP in LAM absent (n=15) n(%)	p-value
Tonus				< 0.001 a
	Normal	10(32.3)	13(86.6)	
	Hypotonic	0(0.0)	1(6.7)	
	Hypertonic	21 (67.7)	1(6.7)	
*Power*				0.745 a
	P ≤2	26(83.9)	12(80.0)	
	P ≥3	5(16.1)	3(20.0)	
	E	4.0±3.6	2.6±3.0	0.175 b
	R	2.1±2.1	3.1±2.6	0.220 b
	F	3.2±3.2	4.9±3.6	0.135 b
Relaxation after maximum contraction in PFM				0.019 a
	Complete	6(19.4)	8(53.3)	
	Incomplete	25(80.6)	7(46.7)	
Electromyography				
	Rest	5.5±2.3	6.8±3.5	0.292 b
	MVIC	55.5±29.4	75.6±50.9	0.210 b
	Endurance	11.7±6.3	18.8±14.8	0.042 b

Data expressed as mean ± standard deviation and n (%);

a Pearson's Chi-square test;

b Mann-whitney test. TP - Trigger point; LAM - Levator ani muscle; PFM - Pelvic floor muscles; MVIC - Maximum voluntary isometric contraction

Regarding the evaluation of the PFM through electromyography, there was a significant difference only for the Endurance variable, and it was observed that women with TPs in the LAM had a lower mean Endurance in the EMG (*P*=.04) (Table 3). When comparing the variables with the presence or absence of Trigger Points in the OIMs, we observed that there was the association between increased vaginal tone and the presence of trigger points in the ROIM (*P=.*01). Regarding comparisons of PFM function between women with or without trigger points in the LOIM, using the PERFECT scale, there was a statistically significant difference for the following variables: P (*P*<.001), E (*P*=.003), R (*P=.*004), F (*P=.*008) e MVIC (*P=.*03) evaluated through electromyography, with lower means being observed in women who had trigger points in the LOIM (Table 4).

**Table 4:** Comparison of the function of PFMs regarding the presence of trigger points in obturator internus muscles

Variables			TP in ROIM present (n=17) n(%)	TP in ROIM absent (n=29) n(%)	p-value	TP in LOIM present (n=25) n(%)	TP in LOIM absent (n=21) n(%)	p-value
Tonus					0.015 a			0.488 a
	Normal		4(23.5)	19(65.5)		11(44.0)	12(57.1)	
	Hypotonic		1(5.9)	0(00.0)		1(4.0)	0(00.0)	
	Hypertonic		12(70.6)	10(34.5)		13(52.0)	9(42.9)	
P					0.115 a			0.001 a
	P ≤2		16(94.1)	22(75.9)		25(100)	13(61.9)	
	P ≥3		1(5.9)	7(24.1)		0(00.0)	8(38.1)	
		E	1.8±2.1	3.7±3.6	0.129 b	1.9±2.9	4.3±3.2	0.003 b
		R	1.7±2.1	2.9±2.3	0.096 b	1.5±1.9	3.5±2.3	0.004 b
		F	2.9±3.3	4.3±3.4	0.241 b	2.6±3.3	5.2±2.9	0.008 b
Relaxing after maximal PFMs contraction					0.149 b			0.695 b
	Complete		3(17.6)	11(37.9)		7(28.0)	7(33.3)	
	Incomplete		14(82.4)	18(62.1)		18(72.0)	14(66.7)	
Electromyogray								
	Rest		5.2±3.1	6.4±2.7	0.233 b	5.7±3.1	6.2±2.5	0.516 b
	MVIC		59.6±53.4	64.2±28.0	0.182 b	55.8±44.6	71.5±28.3	0.037 b
	Endurance		14.6±15.7	13.9±5.8	0.332 b	14.4±13.0	13.9±6.0	0.570 b

Data expressed as mean ± standard deviation and n (%);

a Pearson's qui-square test;

b Mann-whitney test. TP: Trigger point; ROIM: Right obturator internus muscle; LOIM: Left obturator internus muscle; MVIC: Maximum voluntary isometric contraction. PFM: Pelvic floor muscles.

There was a sample loss of 4 participants in the remate sexual function form due to incorrect completion of the questionnaire. In the data obtained, we observed low scores in the FSFI domains by the participants, as well as in the total score (24.7±5.3). When comparing the sexual function between women with or without TP in the LAM, we did not observe a significant difference in any domain, as well as in the total score. This pattern was maintained for TP in the ROIM. However, in comparisons with the LOIM trigger points, there was a statistically significant difference only for the total FSFI score (*P=.*02) (Table 5).

**Table 5:** Sexual function (FSFI) and its association with the presence of TP in the LAM and OIM

FSFI domain	Mean (± SD)	TP in LAM present (n=28)	TP in LAM absent (n=14)	p-value	TP in ROIM present (n=15)	TP in ROIM absent (n=27)	p-value	TP in LOIM present (n=22)	TP in LOIM absent (n=20)	p-value
Desire	40.3±1.0	40.2±1.0	40.5±1.0	00.44 ^a^	40.5±1.04	40.2±1.06	00.40	40.4±0.9	40.2±1.1	00.39 ^a^
Arousal	40.3±1.6	40.6±1.5	30.7±1.6	00.11 ^a^	40.6±1.29	40.2±1.82	00.48	30.9±1.7	40.8±1.3	00.06 ^a^
Lubrication	40.4±1.4	40.6±1.2	40.0±1.7	00.21 ^a^	40.8±1.05	40.2±1.61	00.22	40.0±1.7	40.8±0.9	00.05 ^a^
Orgasm	40.1±1.4	40.4±1.3	30.5±1.4	00.05 ^a^	40.4±1.1	30.9±1.51	00.34	30.8±1.6	40.4±1.0	00.11 ^a^
Satisfaction	30.5±1.3	30.7±1.3	30.3±1.4	00.46 ^a^	30.5±1.3	30.6±1.39	00.95	30.5±1.2	30.6±1.4	00.96 ^a^
Pain	30.9±1.9	30.9±1.8	30.9±2.1	00.92 ^a^	40.3±1.8	30.7±2.00	00.34	30.5±1.9	40.3±1.9	00.19 ^a^
Total score	24.7±5.3	23.1±5.6	25.5±5.1	00.18 ^a^	23.0±9.3	23.9±6.02	00.68	21.3±8.5	26.3±4.5	00.02 ^a^

Data expressed as mean ± standard deviation. Sample loss of 4 participants due to incomplete completion of the questionnaires. TP - Trigger point; ROIM - Right obturator internus muscle; LOIM - Left obturator internus muscle; LAM: Levator ani muscle

## Discussion

Women diagnosed with endometriosis and CPP had changes in pelvic floor function, such as increased muscle tone, muscle weakness and changes in myoelectric activity. In addition, they have Trigger-Points in the PFMs and OIMs, which evidence the diagnosis of Myofascial Pain Syndrome. Women with TP in the LOIM had worse sexual function ratings by the FSFI.

The presence of PFM dysfunction in women with endometriosis and CPP was also observed in the study by Fraga et al.^(17)^, which showed increased tonus in PFM (23/80, 28%), presence of TP (31/80, 38%), pain during vaginal palpation (44/80, 55%), weaker contractions (29/80, 36%) and incomplete PFM relaxation (36/80, 45%) in women with deep endometriosis, both with significant difference compared to the control group without endometriosis. The study by Gyang et al.^(18)^ found increased PFM tone in 22 to 94% of women with CPP.

Montenegro et al.^(19)^ also studied myofascial dysfunctions in endometriosis and revealed that 58,3% (63/108) of women with CPP had Trigger-Points in the PFM. Fraga et al.^(17)^ observed TP in PFMs in 38% (31/80) of women with endometriosis. In a study by Bispo et al.^(20)^, PFM spasms were present in 53% (28/52) of women with endometriosis. These authors also evaluated the OIMs and found an increased prevalence of trigger points in the LOIM. In our study, we found an even higher prevalence of TP in PFM and OIMs, mainly on the left.^(17,19,20)^

The explanation for such predilection in the location of myofascial dysfunctions has already been studied by some authors and may be related to the location of endometriosis lesions. Chapron et al.^(21)^ found that endometriosis lesions, observed in laparoscopic surgery, were predominantly located on the left side of the pelvic wall, preferably on the rectosigmoid. However, other more factors may also be involved, since the location of endometriosis lesions does not always correspond to pain topographies.^(21,22)^

The presence of TP and central pain sensitization can initiate, amplify and perpetuate pain complaints.^(22)^ Women with endometriosis and Myofascial Pain Syndrome are more likely to have hypersensitivity. Chronic pain appears to induce changes in the structure and function of the central nervous system, often leading to central sensitization.^(23)^ Such changes and the presence of TP secondary to the disease can sustain pain and dysfunction even after removal of lesions by surgery and hormonal control.^(11)^ In practice, both central sensitization and myofascial dysfunction are often overlooked in the evaluation and treatment of CPP associated endometriosis.^(22)^ When discussing the importance of palpatory assessment of PFM, Gyan et al.^(18)^ stated that it is often underestimated by physicians. Bispo et al.^(20)^ had already highlighted the small number of articles in the literature that thoroughly described the PFM region of women with endometriosis.

The heterogeneity in clinical assessments of the pelvic floor and the lack of reproducibility in existing digital methods for this evaluation emerge as significant sources of bias in our study. Meister et al.^(12)^ provided recommendations for standardizing the assessment, including patient guidance, using the vaginal clock method for muscle localization, employing single-digit palpation, and quantifying self-reported pain on a scale from 0 to 10. Specifically, muscle palpation should include bilateral LAM and OIMs, as well as observation of the TP and muscle function. We followed these guidelines in our research.^(12)^ However, even with these guidelines, variations in assessment methods and interpretations persist within the field, potentially introducing bias into our findings.

Regarding the function of the PFMs, the study by Fraga et al.^(17)^ also graded the contraction capacity of the PFMs according to the ICS. The authors found that 36% (29/80) of women with endometriosis had a weak pelvic floor. In our study, this percentage was even higher (34/43, 73%).

Changes in PFM function, including reduced muscle contraction capacity and increased tension, are found in women with CPP.^(24)^ We attempted to investigate whether the presence of TP in either the PFM or the OIM could influence PFM function, and although we found a significant difference only for the presence of TP in the LOIM, patients with TP tended to have more dysfunctional PFM. These muscles may appear weak because they actually have higher resting tone, with weakness being related to increased tension.^(25)^

The PFM electrical activity, assessed through surface EMG, is an important parameter to assess PFM resting tonus. The study by Loving et al.^(26)^ also evaluated, on surface EMG, the tonic activity at rest for 60 seconds in women with CPP. However, they found an activity at rest of 1.90 μV, while, in our study, the average found was 6 μV. In the study by Loving et al.^(26)^, 45% (11/24) of women with CPP had an average greater than 2 μV. The two studies found, in addition to an increase in PFM resting tone, a decrease in relaxation capacity in women with CPP assessed by digital palpation and EMG. We did not find studies related to CPP and endometriosis that discussed MVIC and Endurance.^(26)^

The presence of TP can be related to a lower capacity for relaxation of PFMs, since TPs are hypersensitive areas that compromise the ability of the muscle to contract and relax.^(7)^ We found, in our study, that women with endometriosis and CPP who had TP in the LAM had a lower capacity for relaxation. Fraga et al.^(17)^ found incomplete relaxation in 45% (36/80) of the women, Loving et al.^(26)^ observed partial relaxation in 66% (16/24) and, in the present study, we observed incomplete relaxation in 30% (14/46). We draw attention to the women who showed absence of PFM relaxation, present in 24% (11/46) of participants in our study, in 12% (3/24) in the study by Loving et al.^(26)^ and in 0% (0/80) in the study by Fraga et al.^(17)^. We also emphasize that, in our study, we found a higher frequency of TP in PFM (31/46, 67%) than data previously reported in the literature.^(17)^

The PFMs of the women with CPP suffer constant overload resulting from postural changes in an attempt to relieve pain. This increase in tension and sensitivity can intensify dyspareunia.^(5)^ In endometriosis, MPPS can impair urinary, bowel and sexual function, as well as being associated with a significant reduction in quality of life.^(9,27)^ In addition to dyspareunia, the women with endometriosis may have other sexual complaints, such as loss of lubrication, arousal and desire.^(28)^ Anticipation and fear of pain can cause such symptoms, in addition to increased AP tone, resulting in a change in sexual function and satisfaction.^(29)^

A study carried out by Evangelista et al.^(28)^ through the analysis of Brazilian studies, investigated the female sexual function, also through the FSFI in women with endometriosis, and concluded that the FSFI score of 26,5 can be used as an optimized cut-off point between normal sexual function and sexual dysfunction. In our study, we showed that women with endometriosis and CPP had a mean FSFI score of 24,7 (SD 5,3), as well as the finding of Evangelista et al.^(28)^, who found a mean of 23,4 in the FSFI, suggesting the presence of sexual dysfunction in women with endometriosis.

The women with endometriosis and CPP had a lower total FSFI score When they had Trigger Points in the LAM and OIMs. Regardless of the presence of TP, women had a total score less than 16.5, a factor that may have influenced the lack of significant difference between groups. A significant difference was observed only for the total FSFI score in relation to the presence of trigger points in the LOIM, indicating worse sexual function in these women. This finding may be related to greater pain symptoms, reinforcing the importance of evaluating myofascial disorders.

The limitations of our study are the small number of participants, as well as the lack of comparison with a control group without the disease, which may limit the analysis of some associations. Another important limitation of this study is the absence of sample size calculations. The failure to conduct a proper sample size calculation can undermine the study's statistical power and the ability to draw robust conclusions from the data. Additionally, we did not analyze the anatomopathological results of the women who underwent laparoscopy and their relationship with myofascial dysfunctions to try to explain the higher frequency of TP on the left. We can highlight as strong points of this study the investigation of the presence of the MPPS and its relation with the PFM and sexual function; the standardization of the assessment performed by only one physiotherapist experienced in diagnosing PFM disorders; the association of two evaluation methods (digital palpation and electromyography) and the use of the FSFI, which is a standardized and widely used questionnaire to assess female sexual dysfunctions.

The management of CPP associated with endometriosis remains challenging, as its multifactorial origin can lead to the persistence of symptoms even after surgical removal of endometriotic lesions.^(11)^ A multidisciplinary approach is necessary for a more effective assessment and treatment, as women with endometriosis also present myofascial dysfunctions associated with the CPP condition.

## Conclusion

The presence of MPPS may be associated with impairments in PFM function. Regarding sexual function, the diagnosis of endometriosis and CPP is already related to poorer sexual function, regardless of the presence of myofascial trigger points. Given these dysfunctions, which are related to PFM function and sexual function, the physiotherapist should be an integral part of the multidisciplinary care throughout the therapeutic process. This allows for standardized and regular assessments, as well as more effective treatment for women with endometriosis and CPP.
